# Amlodipine as a Suggested Cause of Yellow Nail Syndrome: A Case Report

**DOI:** 10.7759/cureus.29396

**Published:** 2022-09-21

**Authors:** Hasan Al Houri, Heba Al-Tarcheh, Ousama Zghaier, Salloum Salloum, Ahed Haj Ibrahim, Moudar Kouli

**Affiliations:** 1 Department of Internal Medicine, Syrian Private University, Damascus, SYR; 2 Department of Internal Medicine, Al Assad and Al Mouwasat University Hospitals, Damascus, SYR; 3 Department of Pulmonary Medicine, Al Assad University Hospital/Damascus University, Damascus, SYR; 4 Department of Cardiology, Martyr Bassel Al-Assad Heart Hospital, Damascus, SYR; 5 Department of General Medicine, Damascus University, Damascus, SYR

**Keywords:** nails, dermatology, lymphedema, amlodipine, yellow nail syndrome

## Abstract

Yellow nail syndrome (YNS) is a rare disorder initially described in 1964. It is characterized by a classical triad: yellow nails, lymphedema, and respiratory manifestations. We present a 71-year-old woman who presented with progressive dyspnea. Medical history includes hypertension treated with amlodipine. Examination showed bilateral lower extremity non-pitting edema, yellowish discoloration of nails, and bilateral pleural effusion. Thoracentesis demonstrated chylous effusion. The presumptive diagnosis was YNS. Assuming amlodipine as a cause of interstitial edema, it was stopped, and the symptoms improved gradually. After two months, amlodipine was restarted externally, and the dyspnea relapsed. Amlodipine was discontinued again. After two years of amlodipine cessation, the patient remained well without symptoms. The progression and resolution of symptoms point to amlodipine as a suggested cause of YNS. Paying attention to the prescribed drugs was the key to diagnosing and resolving serious complications.

## Introduction

Yellow nail syndrome (YNS) is a scarce entity initially described in 1964 [1]. It is characterized by a classical triad consisting of yellow-green dystrophic nails, lymphedema, and respiratory manifestations [1,2]. The pathogenesis of YNS remains obscure. However, it is considered secondary to functional lymphatic drainage abnormalities [2]. Amlodipine is a long-acting calcium channel blocker used as an antihypertensive medication [3]. To the best of our knowledge, this is the first case that reports amlodipine as a suggested cause of yellow nail syndrome.

## Case presentation

A 71-year-old non-smoker female presented with progressive exertional dyspnea within the previous four months with a productive cough. The patient denied the presence of fever, weight loss, night sweating, and anorexia.

The patient’s past medical history includes hypertension treated with amlodipine 5 mg daily for the last four years and chronic sinusitis, and her family history is insignificant. On admission, vital signs were normal except for oxygen saturation (SaO2) of 91% and a respiratory rate (RR) of 23 breaths per minute.

**Figure 1:**
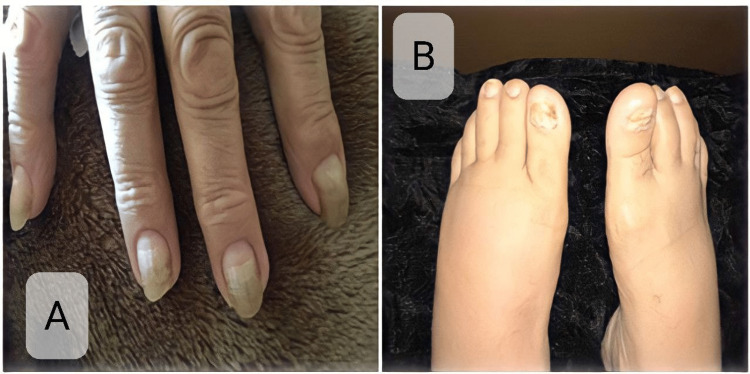
Yellow nail discoloration in the fingernails (A) and toenails (B).

General examination exhibited facial edema, bilateral lower limb non-pitting edema, and yellowish discoloration of the fingernails and toenails noticed by the patient three and half years ago (Figure 1).

Physical examination revealed bilateral diminished lung sounds. The patient was treated with antifungals topically and systematically for six months despite a negative fungal culture.

**Figure 2:**
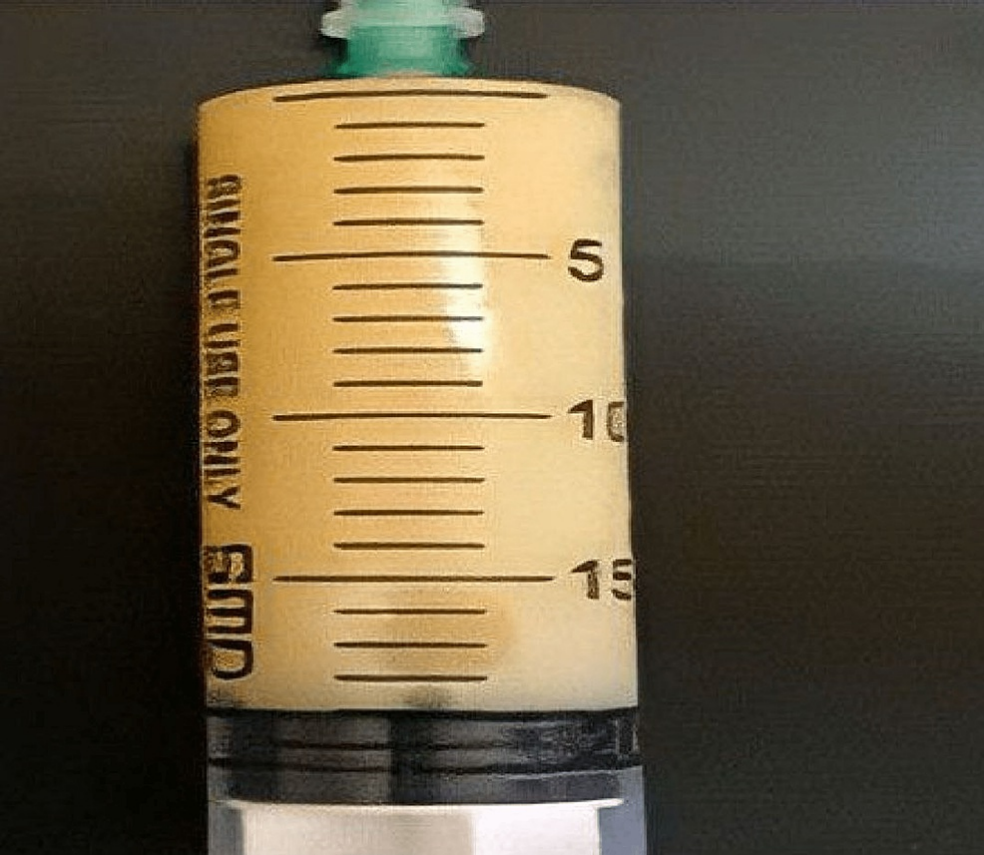
Pleural fluid drained from the right side: milky appearance suggests chylous effusion.

Blood tests were normal. Chest X-ray (CXR) revealed bilateral pleural effusion and interstitial infiltrations. Rhinosinuses CT-scan showed increased maxillary sinuses mucous membranous thickness. Chest CT scan showed bilateral pleural effusion more significant on the right side and interlobular thickenings in the lung bases. Echocardiography was normal. Right side thoracentesis demonstrated chylous effusion (Figure 2, Table 1).

**Table 1:** Biochemistry of the Pleural effusion aspiration. Abbreviations: WCC: white cell count, L/N: Lymphocytes/Neutrophils, RBC: red blood cells, TP: Total protein, LDH: Lactate dehydrogenase, GLU: glucose, CHOL: cholesterol, TG: Triglycerides, mm^3^: millimeter cubic, g/dl: gram/deciliter, u/l: unit/liter. *Patient serum LDH 192 u/L; **Patient serum Glucose 87 mg/dl

	Appearance	WCC	L/N	RBC	TP	LDH	GLU	CHOL	TG	PH
	White Chylous	2300/mm^3^	90%/10%	650/mm^3^	3.4 g/dl	212 u/L	144 mg/dl	47 mg/dl	256 mg/dl	8
Normal Range	Clear	<1000/mm^3^		None seen	1-2 g/dl	<50% plasma LDH*	Similar to Plasma GLU**	135-250 mg/dl	<75 mg/dl	7.60-7.64

Pleural fluid microbial and tuberculosis tests returned negative. Abdominal, pelvic, and breast ultrasounds were normal. 1100 ml of chylous were drained. Because of the rapidly recurred effusion, talc-induced pleurodesis for the right thorax was indicated. It was repeated due to relapse, done by a consultant in thoracic surgery. The patient was monitored for a week in the hospital post-operation with only minimal improvement. The lymphoscintigraphy was not available in the hospital; therefore, according to the three cardinal signs: yellow nail discoloration, pleural effusion, and lymphedema, the presumptive diagnosis was yellow nail syndrome.

Treatment with 1200 IU/day of vitamin E orally was initiated. Even after several months, CXR showed bilateral pleural effusions. Dyspnea persisted despite pleural fluid drainage. Assuming amlodipine as a cause of interstitial edema, it was stopped. Ten days later, the symptoms improved and the patient was able to do activities of daily life without dyspnea. Her SaO2 was 96%. After three weeks, a follow-up CXR showed clear lung fields with pleural thickening. The patient was discharged well; however, two months later, the dyspnea relapsed. The patient declared starting a new drug five days ago prescribed by her doctor due to uncontrolled hypertension. Surprisingly, the prescribed drug contained amlodipine: EXFORGE© (amlodipine - valsartan) (Novartis AG, Basel, Switzerland). Amlodipine was discontinued for the second time and the dyspnea improved after two weeks.

Follow-up

**Figure 3:**
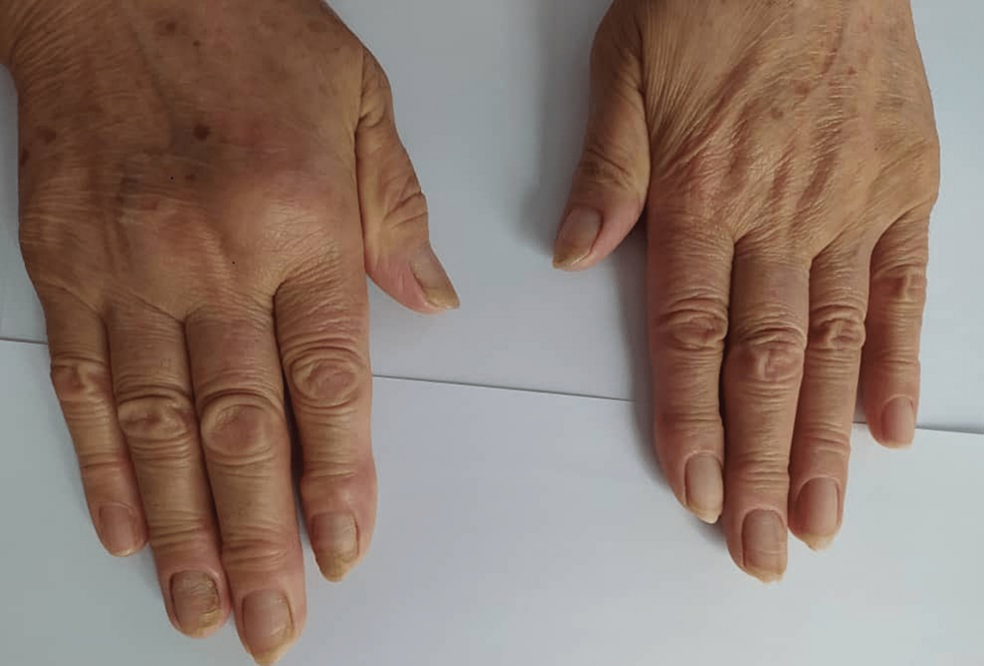
Improvement in the nail color after two years of Amlodipine cessation. Nail growth was slow in comparison to normal individuals (0.1-0.25 mm per week in yellow nail syndrome versus 0.5-2 mm per week in normal population).

After two years of amlodipine cessation, the patient is still doing well without symptoms or unanticipated events. Vital signs, clinical evaluation, and CXR are normal. The nails recovered noticeably (Figure 3).

The patient was able to function as she used to without assistance. No other interventions were required after two years of follow-up.

## Discussion

Yellow nail syndrome (YNS) is a rare condition with less than 400 cases in the medical literature [1]. The YNS diagnostic triad is yellow nail color, pulmonary disease, and lymphedema [1,2]. The syndrome is heterogeneous and the diagnosis requires two manifestations. The full triad was mentioned in 27%-60% of cases [4]. Nail discoloration involves both fingernails and toenails and is essential for diagnosis. It is described as pale yellow to dark green thick nails with increased curvature. Pulmonary disease is frequent in YNS (56%-71%) [4]. Respiratory findings include pleural effusion, recurrent pneumonia, bronchiectasis, and sinusitis [4]. The effusions are mostly exudative (95%) [5], while chylothorax occurs only in 22% of cases [5]. Non-pitting lymphedema occurs in one-third of cases. It may be general in the face and limbs, but dominantly in the legs, which may predispose to cellulitis. Lymph accumulation, fibrosis, and adipose tissue explain the edema chronicity and irreversibility.

In a study of 17 YNS patients, lymphoscintigraphy demonstrated lymphatic insufficiency [6]. Lymph uptake and transport were reduced by (41%-44%) in axillary and ilioinguinal lymph nodes [6]. The lymphoscintigraphy was not applied in our hospital. In chylothorax effusions, lymphatic vessels were dilated in the parietal pleura [7]. Aging can participate in lymph drainage dysfunction without anatomical problems. [6] In addition, increased microvascular permeability leads to protein leak and edema. YNS may be associated with malignant and non-malignant cases including immunodeficiencies [8]. Some drugs such as D-penicillamine, gold, thiol-containing drugs, and bucillamine can cause nail discoloration, which resolves after discontinuation [8]. Nail infections and mycosis should be ruled out.

Amlodipine is a dihydropyridine calcium channel blocker (CCB) used for hypertension and angina [3]. Amlodipine inhibits the opening of voltage-gated L-type calcium channels [3]. Common side effects are peripheral edema, headache, fatigue, and palpitation. One study reported pulmonary edema and pleural transudate effusion after taking 120 mg of nifedipine with 5 mg of amlodipine [9]. While in this case, the patient used 5 mg amlodipine only. The patient’s symptoms improved ten days after discontinuation and returned after resuming it. This is highly suggestive of amlodipine being the contributory agent. CCBs induce pericapillary vasodilatation influencing lymphatic vessels and drainage leading to non-cardiogenic pulmonary edema and pleural effusion [10]. Amlodipine action in addition to unknown functional or anatomical mild abnormalities may reproduce the full syndrome.

 The prognosis of YNS is generally favorable. Treatments include oral vitamin E, topical steroids, and topical and systematic antifungals [8]. Decorticating, pleurectomy, pleuroperitoneal shunt, and talc pleurodesis were used in recurrent or large effusions [7]. Octreotide and triglyceride-reduced diet are conservative treatments in chylothorax [7].

## Conclusions

YNS is considered a complicated case requiring multiple medical consultations. The progression and resolution of symptoms point to amlodipine as a cause of the disease. Paying attention to the prescribed drugs was the clue in making the diagnosis and resolving serious complications.
